# Design of a piezoelectrically actuated hydrocephalus shunt valve

**DOI:** 10.1007/s11517-023-02822-1

**Published:** 2023-03-30

**Authors:** O. Salih, M. Messina, D. Al-Jumeily

**Affiliations:** 1 Engineering and Technology Research Institute, Liverpool John Moores University, Liverpool, UK; 2 Mechanical Engineering Department, Liverpool John Moores University, Liverpool, UK; 3 School of Computer Science and Mathematics, Liverpool John Moores University, Liverpool, UK

**Keywords:** Actuation, Hydrocephalus, Mechatronic valve, Piezoelectric, Shunt, Ultra-sonic motor

## Abstract

Hydrocephalus is currently managed by using traditional mechanical passive shunts. Due to their nature, these shunts have fundamental shortcomings including an increase in patient shunt dependency, absence of fault detection, and over drainage due to lack of shunt proactivity. There is a scientific consensus that the way forward to address these issues is through what is termed a smart shunt. The core component of this system is the mechatronic controllable valve. A design of a valve which utilises both the passive nature of the classical valves and the controllability feature of the fully automated valves is presented in this paper. The valve consists of a fluid compartment, a linear spring, and an ultrasonic piezoelectric element. The valve is designed to operate on a 5 V supply, to drain up to 300 mL/h, and it has an operational range between 10 and 20 mmHg. The design produced is considered feasible as it takes into consideration the multiple operation conditions associated with such an implanted system.

**Graphical Abstract:**

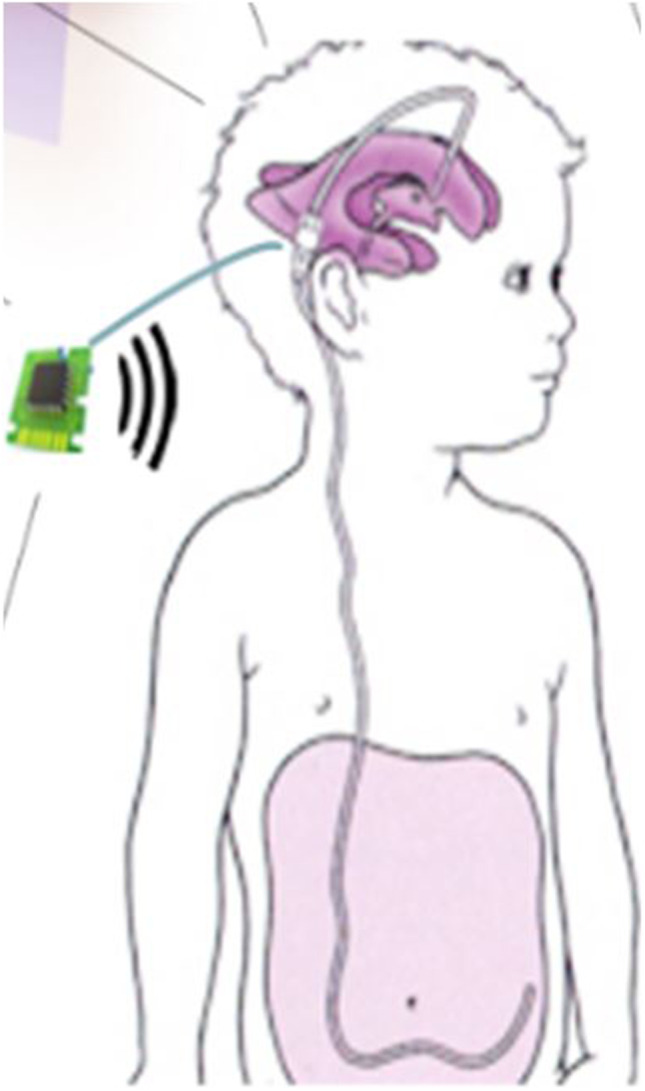

## Introduction

Hydrocephalus (HC) is defined as the disorder resulting from “the dynamic imbalance between the production and absorption of cerebrospinal fluid (CSF) leading to enlarged vortices” [1]. It is the result of the increased CSF amount in the ventricles caused by a disruption in its flow, absorption, or formation process [2, 3]. Hydrocephalus is managed by using CSF shunts which regulate CSF flow using a shunt’s valve. The two main types of these valves are the first-generation fixed differential pressure valves (DPVs), and the Programmable (adjustable) valves [4]. Both valves’ operation is based on the difference between the intracranial pressure (ICP) and the drainage site pressure [5, 6]. However, with the adjustable valves, there is the option of adjusting the opening pressure externally using magnetic devices [7]. It must be noted that once the adjustable valve’s opening pressure is set, they behave exactly like DPVs [8]. These valves have been developed in the 1980s and have been in use since [9, 10]. There are several drawbacks to the currently used shunting systems including the fact that (i) it does not take into consideration the changes in the dynamic behaviour of ICP which vary, not only from one patient to another but also for the same patient depending on age, health, and other elements [3, 5, 6, 9]. (ii) The current shunting system tends to encourage the patient shunt dependency to increase with time due to the lack of personalisation [3, 10]. (iii) The system lacks proactivity and does not recognise the rise in ICP due to normal events such as coughing and sneezing which leads to unnecessary drainage (i.e. over-drainage) [11]. The drained CSF could take hours to be re-produced again [12]. (iv) Furthermore, any shunt malfunctions cannot be detected until they manifest clinically as symptoms. This can be life-threatening based on the type of malfunction and the patient’s condition. Current statistics indicate that 30–40% of shunts fail within the first year of use, 50% fail within the first two years, and 90% of them fail after 5 years [13–16]. According to the 2017 UK shunt registry report [17], there were 3000 shunt operations in the UK in that year, 1660 for pediatric patients and 1400 for adults. Of pediatric operations, 66.5% were for shunt revision as appose to primary first-time installation, while 47% of the adult operations were for shunt revisions.

Most studies on developing new shunts are focused on the use of membranes as appose to traditional valve techniques such as the ball-in-cone valve [12–16]. It is understandable as this shunt type results in a more pressure-sensitive valve and a much more compact system. However, these designs still have the same characteristics as currently used valves and are thus prone to the same fundamental issues. The solutions discussed in the literature are all based on the concept of a smart shunt. A shunt that is controllable based on multiple sensory inputs. This work provides the designing process of a proposed mechatronic shunt valve which is the core component of a smart shunt.

## Method

Three key factors are put in place for the selection of the mechatronic valve layout and its components. The first one is size and compactness which relates to the space issue as the valve needs to be as small as possible since it is implanted under the skin in the area above the ear. The valve also needs to be designed so that all components can fit in a single housing for it to be compact. The second factor is component suitability which relates to materials bio and MRI compatibility. Other issues are related to patient inconvenience such as noise (i.e. from friction) as the device is in the patient’s head. The third factor is power consumption as the valve is intended to be a long-term management system. Therefore, it is important to keep the power draw at a minimum.

**Fig. 1:**
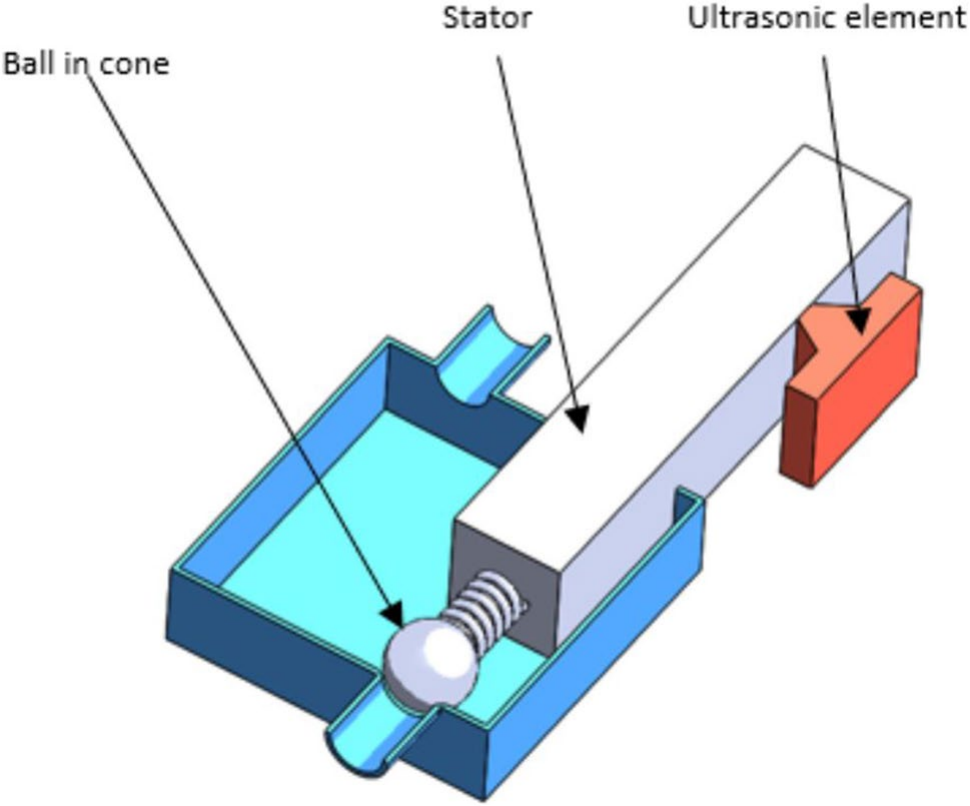
Proposed mechatronic valve layout and components. Consists of an ultrasonic element supplying movement to the preloaded stator which controls the ball in a cone mechanism

The layout and components selected are shown in Fig. 1. The use of a spring and a ball system controlled by an ultrasonic piezoelectric element provides minimum battery usage as the motor only comes online when the valve opening pressure needs to be changed. Furthermore, the ultrasonic element is self-locking when it is offline. The ultrasonic element also operates on low voltage (3–5 V). This is ideal since it will be powered by an implanted battery. The system is also MRI-compatible since there are no magnetic components to cause interference.

The design process consists of a CFD simulation to understand the fluid forces during operation. The second part is designing the spring controlling the open/close status of the valve. The third part is designing the ultrasonic element controlling the spring.

### CFD analysis

Several numerical methods and techniques are used to approach CFD modelling and analysis. These methods are used to approximate a solution of the flow describing equations. These equations are termed “Navier–Stokes” equations. They are used to describe the motion and behaviour of viscous fluids. Newton’s second law is employed in those equations and applied to fluid behaviour. It takes the sum of viscous terms, fluid stress, and a pressure term [18].

1 $$\frac{\partial\rho}{\partial t}+\frac{\partial\rho\;u_i}{{\partial x}_i}=0$$

2 $$\frac{\partial \rho {u}_{i}}{\partial t}+ \frac{\partial \rho {u}_{i} {u}_{j}}{{\partial x}_{j}}=- \frac{\partial P}{{\partial x}_{i}}+ \frac{\partial (\mu \frac{\partial {u}_{i}}{{\partial x}_{j}})}{{\partial x}_{j}}+ {S}_{{M}_{i}}$$

Equations 1 and 2 are known as the “continuity” equation and the “momentum” equation, respectively, where $\mu$ is the dynamic viscosity (Pa.s) and ${S}_{{M}_{i}}$ is a term that represent external effects. CSF is extremely similar to water and thus water properties are used. First Reynold’s number is calculated from:

3 $$Re= \frac{\rho V D}{\mu }$$

where *V* is the velocity (mm/s) and *D* is the catheter diameter (mm). Normal CSF volume production in hydrocephalus patients is usually around 2000 ml/day [19]. The model used has a 1 mm pipe inside diameter for both the inlet and outlet as that is the average inside diameters of proximal and distal catheters [20]. The resulting Reynold’s number clearly indicates a laminar flow. However, it is not low enough for the flow to be considered a creeping flow which usually occurs at *Re* > 1.

The valve is to be designed for 11 pressure points from 10 to 20 mmHg with 1 mmHg intervals. It is extremely important to take into consideration the effects of hydrostatic pressure. As a result, simulations will be run for those 11 points under both default conditions and conditions where the hydrostatic pressure is in effect. These 11 points are intracranial pressure points. However, the simulation uses pressure at the inlet point of the valve. This means that pressure losses due to friction in the proximal catheter need to be taken into consideration. The equations are as follows [21]:

4 $${P}_{friction}= \frac{128 \mu L Q}{\pi {D}^{4}}$$

5 $${P}_{h}= \rho g h \mathit{sin}\left(\Psi \right)$$

6 $${P}_{inlet}= ICP- {P}_{friction}$$

7 $${P}_{h-inlet}= ICP- {P}_{friction}+ {P}_{h}$$

where ${P}_{friction}$ is the pressure loss due to friction (Pa), ${P}_{h}$ is the hydrostatic pressure, ${P}_{inlet}$ is the pressure at the valve inlet, and ${P}_{h-inlet}$ is the pressure at the valve inlet in the standing position. $L$ is the proximal catheter length (mm), $g$ is the gravity acceleration (m/s/s), $h$ is the column of fluid elevation (mm), and Ψ is the angle of that column. Since the shunt is placed near the ear to the back of the head, the length between the shunt and the proximal catheter inlet is $h$ which is approximated as 300 mm. The hydrostatic pressure is at its highest at the upright position and thus Ψ is taken as 90 degrees. This means that the total pressure design points are 22 as each pressure point has two conditions, the default condition and the under hydrostatic pressure condition.

**Fig. 2:**
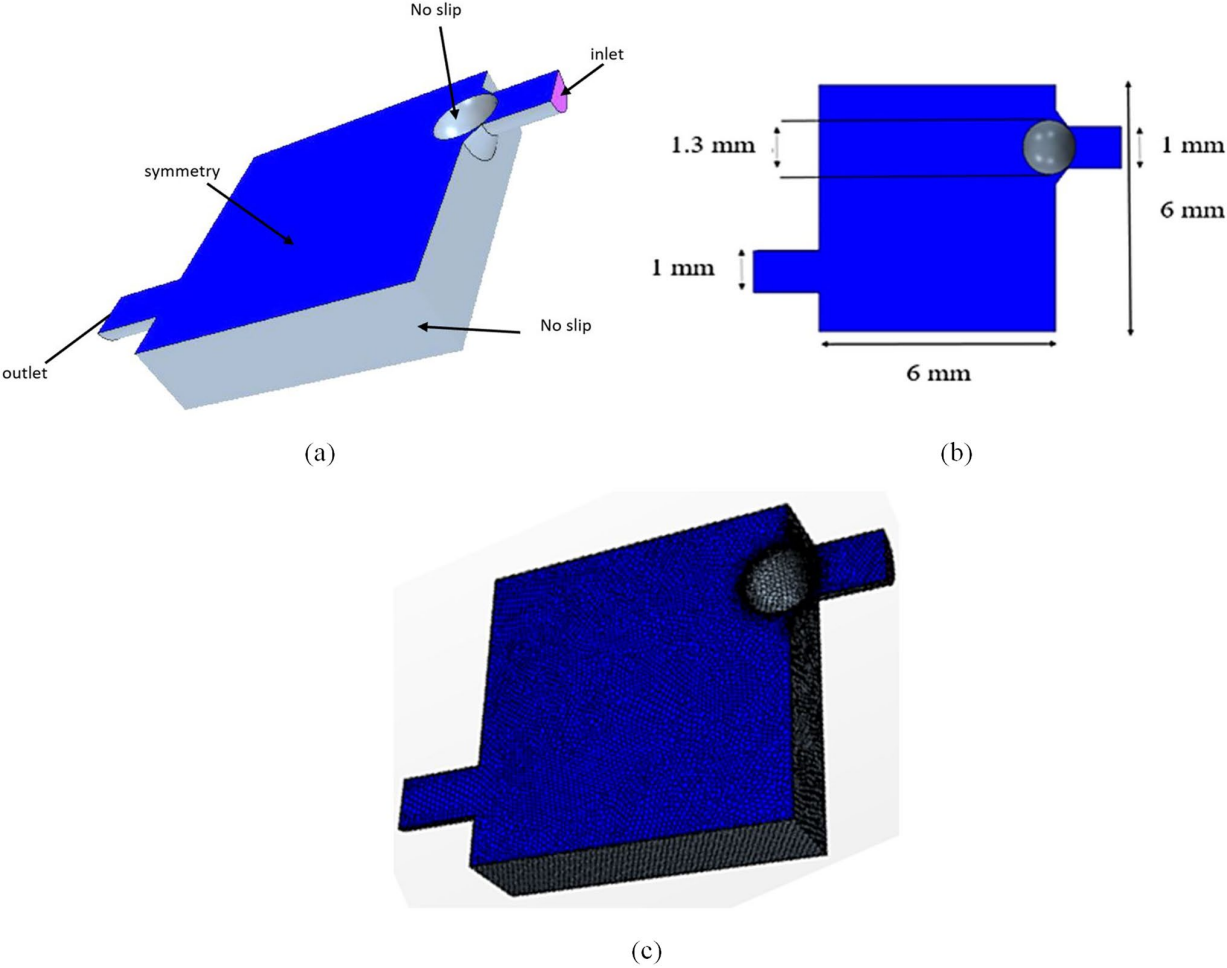
Valve CFD model. **a** Model Boundary conditions. **b** Model Mesh. **c** Model dimensions. No-slip boundary condition indicates zero velocity relative to the boundary. Since only half the model is used a symmetry boundary condition is used to mirror that half

The model boundary conditions are shown in Fig. 2a. All walls including the ball are set to no-slip–zero velocity relative to the boundary–boundary conditions. The inlet boundary condition is inlet pressure for each of the 22 pressure points. The outlet boundary is the distal pressure. The distal catheter drains the CSF into the heart’s right atrium where the pressure is around 6 mmHg (799.9 Pa) [22, 23]. As the flow is laminar, minimum attention was given to refining wall mesh areas. However, as seen in Fig. 2b, a fine mesh was used near the ball area due to the small size of that area. The total number of cells used is 176,000.

### Spring design

The finite element method (FEM) is a numerical technique used to solve the partial differential equations (PDEs) used to describe a physical phenomenon. For most problems, an approximation of these PDEs into numerical equations is required. The solution of these numerical equations is considered an approximation to the real solution of the PDEs [24]. Initial spring dimensions were first produced using general design equations. This was used in the simulation as the first iteration in the process of the design.

**Fig. 3:**
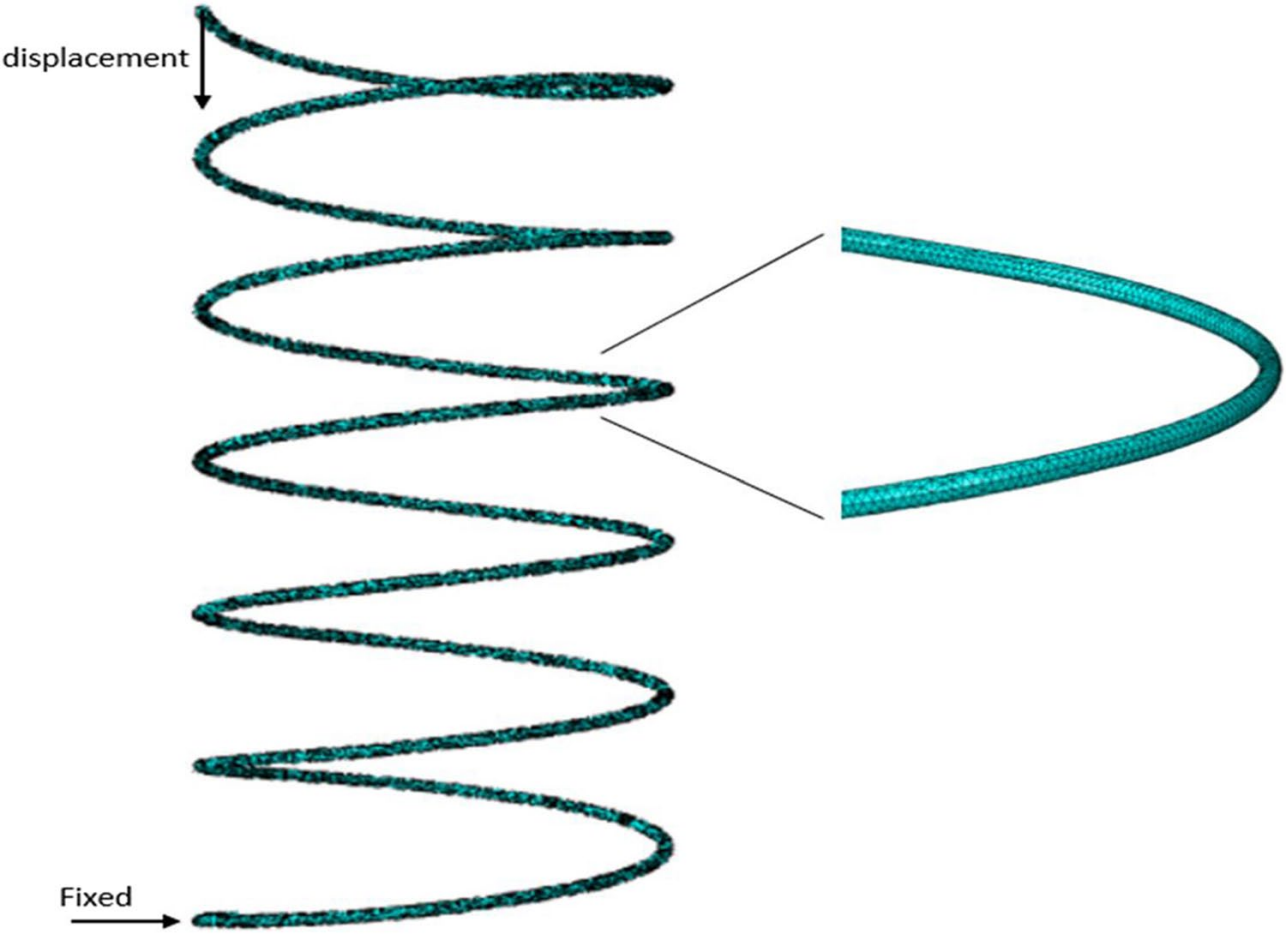
Spring FEA model. Displacement and fixed boundary conditions are used

Two models are built. One for maximum displacement at maximum force at 20 mmHg, and one at minimum at 10 mmHg. The boundary conditions used are a fixed end surface of one side of the spring and applying displacement on the other end surface. Based on the initial design, a displacement of 2.22 mm and 0.22 mm is applied to the spring to simulate maximum loading conditions at 20 mmHg and minimum loading conditions at 10 mmHg, respectively. A total of 167,000 elements are used in the simulation. Figure 3 showcases boundary conditions and meshing quality used. The material properties used are those of Magnesium AZ91E-T6. The free length, wire diameter, and outer diameter of the model are 4.22, 0.06, and 1.8 mm, respectively.

### Ultrasonic element design

**Fig. 4:**
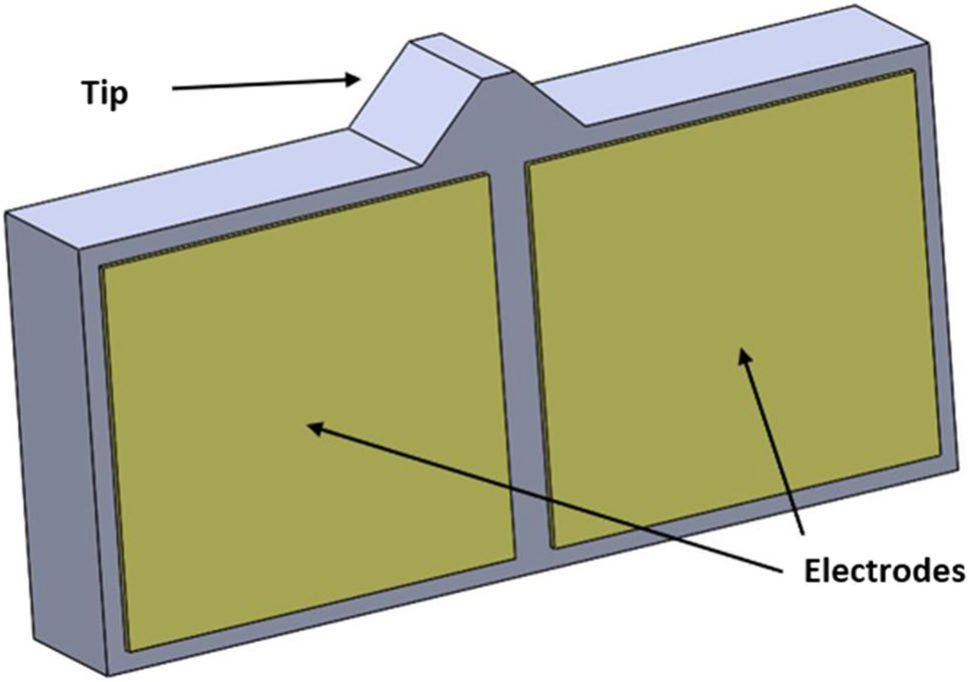
Piezoceramic element model. The two electrodes are used for the voltage input. Voltage is applied to each one separately to get a different movement direction

The operation of the ultrasonic motor depends on mechanical resonance. The ultrasonic element—rotor—is excited using a high-frequency current and with the right frequency excitation, the element will be under resonance. The resulting deformation is then harvested through friction to induce displacement to the stator. Figure 4 presents the model of the motor. The element is asymmetrically excited by applying a voltage through one of the electrodes. This results in deformation that gives motion—utilizing tip and stator friction—in one direction. The main design process for these motors is to conduct an FEA analysis on the motor. A modal analysis is conducted to understand and visualise different modes of excitation and which one of these modes is to be used. This is considered a frequency extraction step. The other step is to simulate the partial excitement of the element by applying a voltage at the extracted frequency.

#### Modal analysis

**Fig. 5:**
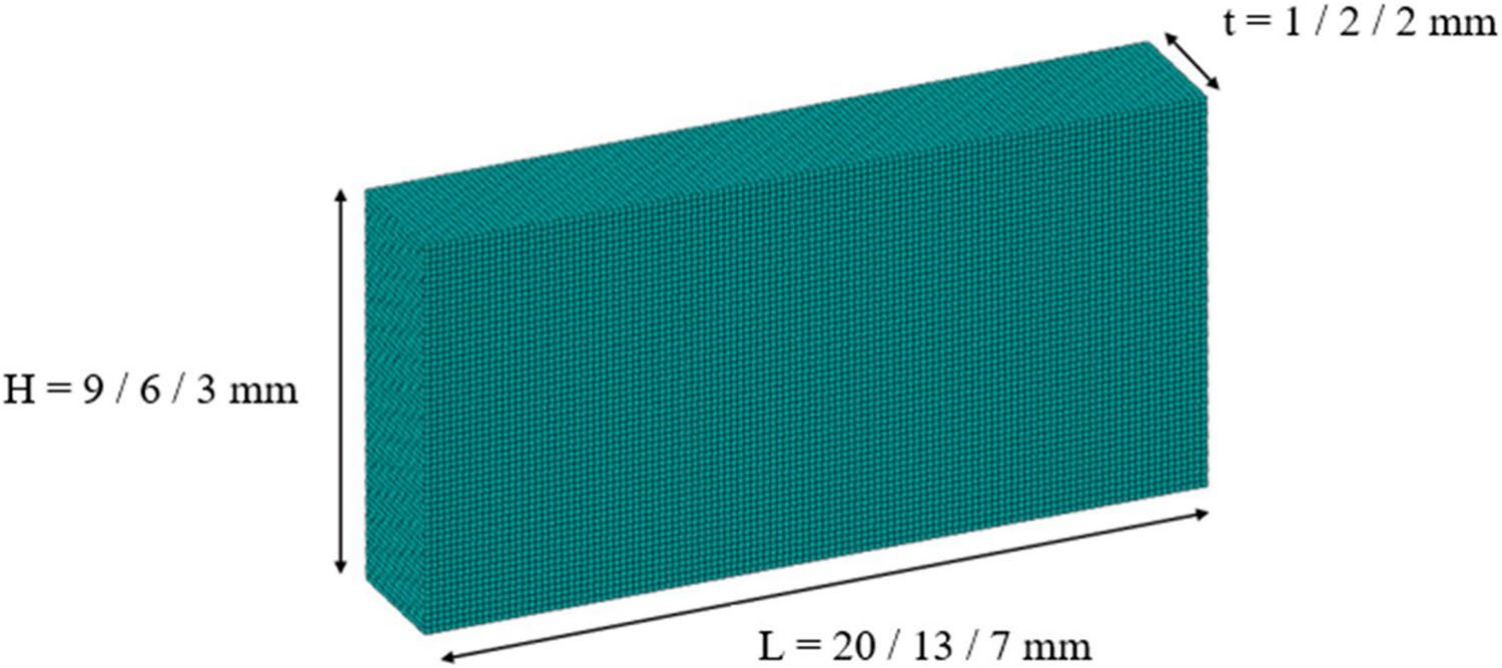
Piezoceramic frequency analysis model. The model showcases the 3 sheet sizes respectively

Acoustic waves spread as compression waves in solid bodies. In small enough bodies, this can result in creating standing waves (two waves with the same amplitude and frequency moving in opposite directions). However, in bodies with two dimensions of the same order such as metal sheets, two-dimensional standing waves are observed. These waves mirror each other on each axis which is necessary to ensure that the element will produce the same deformation when partially excited on each side and hence produce a bi-directional displacement. These mode types are termed E*(K,I)* where E refers to “extensional” as these modes cause planar extension. *K* and *I* are the number of half wavelengths in the X and Y direction, respectively [25, 26]. For a piezoceramic plate with a length ($L$) in the X direction, height ($H$) of 0.5 $L$ in the Y direction, and polarisation in the thickness ($t$) in the Z direction two E(3,1) modes are observed. These modes consist of 2-d standing waves. The one in the X-direction has 3 half wavelengths ($L= 3{\lambda }_{x}/2$), and the one in the Y-direction has a one-half wavelength ($H={\uplambda }_{y}/2$). The mode shape can be described as [27]:

8 $${U}_{x}\left(x,y,t\right)=- A\mathit{sin}\left(\frac{3\pi }{L}x\right) \left(\mathit{cos}\left(\frac{\pi }{H}y\right)-1\right)sin(\omega t)$$

9 $${U}_{y}\left(x,y,t\right)=B\left(1\pm C\mathit{cos}\left(\frac{3\pi }{L}x\right)\right)\mathit{sin}(\frac{\pi }{H}y) sin(\omega t)$$

where ${U}_{x}$ and ${U}_{y}$ are displacements in the X and Y directions. $A$, $B$, and $C$ are geometrical and material functions. A modal analysis is conducted to find the resonance frequency of three piezo elements. Their dimensions are 7 × 3 × 1, 13 × 6 × 2, and 20 × 9 × 2 mm as shown in Fig. 5. The first 100 modes of resonance are set to be extracted. The total number of elements used is 140,000 elements. The piezoceramic material used is PTZ-5H. It has better piezoelectric properties compared to other piezoceramics.

#### Dynamic analysis

The next step after confirming the E(3,1) mode frequency is to simulate the operation of the piezo motor through partial excitation. As seen above in Fig. 4 two electrodes are used to control the piezo motor. A high-frequency current must be simulated through the piezo element to understand what type of reaction is to be expected. The 13 × 6 × 2 mm sheet is used as it is smaller which makes it more appropriate for this application.

**Fig. 6:**
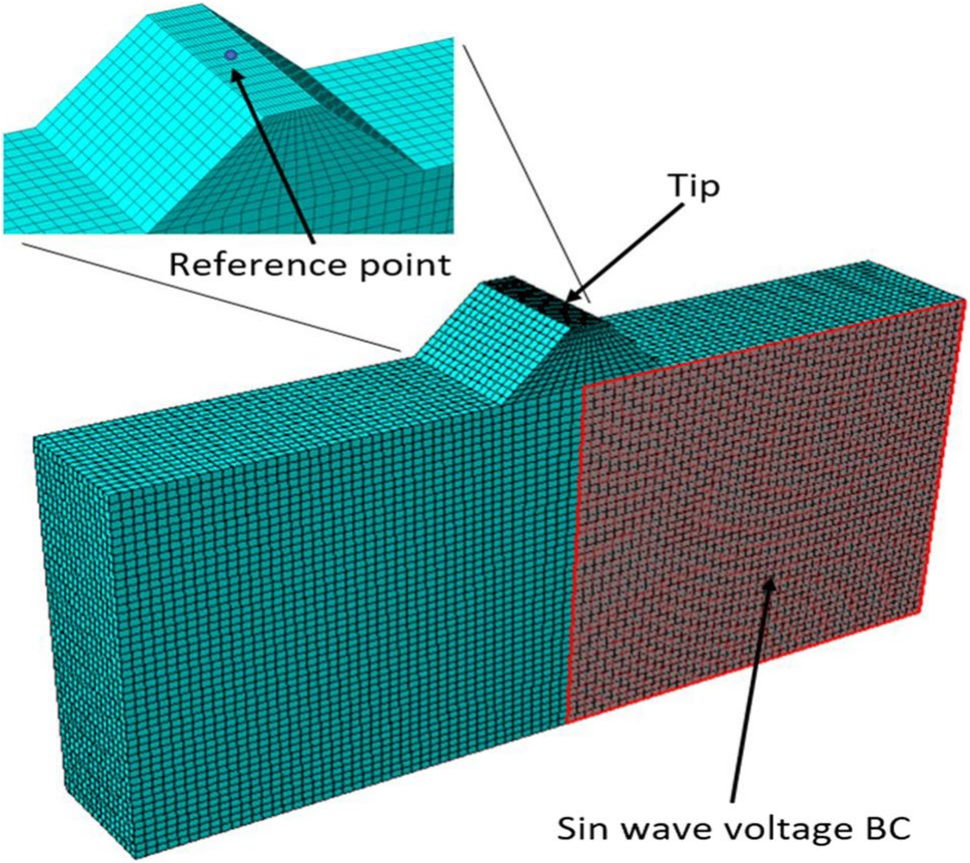
Piezo element partial excitement model. The entire second side of the model is grounded with zero voltage

A tip is added at the midpoint of the model as shown in Fig. 6. The tip is the part that will be in contact with the stator. It is made of aluminium oxide as it is a material with a high friction coefficient. A sin wave voltage of 3.5 V is set as a boundary condition on half of the model face on one side. The frequency of the sin wave is the E(3,1) resonance mode which is 294.8 kHz. This frequency is used as it is the E(3,1) mode found during the modal analysis as will be shown in the results section. The other side voltage is set to ground across the entire face. The total number of elements used is 60000. The time period on the dynamic simulation is 1.7E-5 s. This is enough time for 5 voltage cycles ($5*1/f$).

## Results

### CFD analysis results

**Fig. 7:**
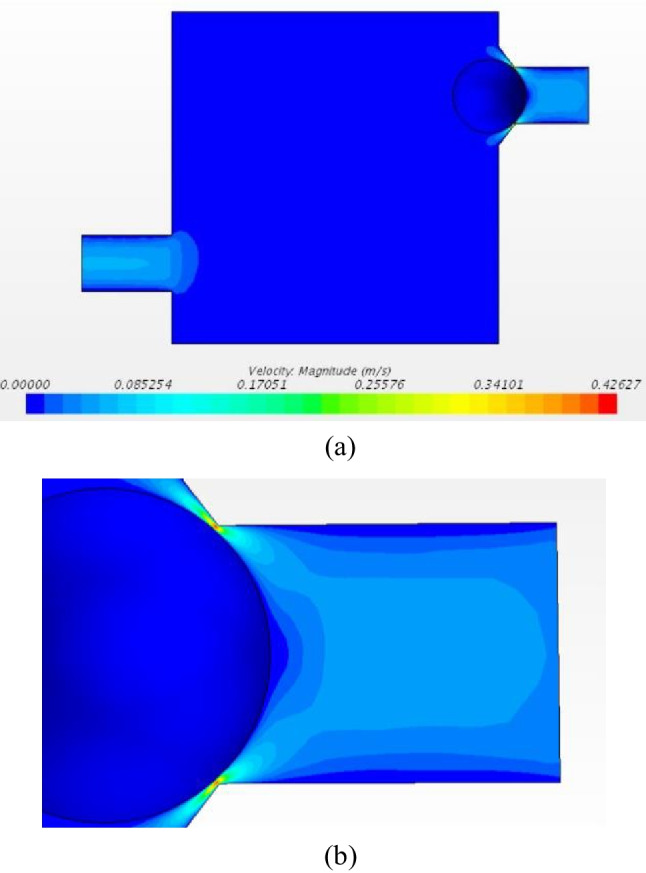
CFD simulation results at 10 mmHg. (**a** Velocity profile. **b** Zoomed-in velocity profile

Figure 7 shows the velocity profile at the minimum pressure point of 10 mmHg. High-velocity values are consternated in the area near the ball on the inlet side. Force on the ball as a result of both fluid pressure and shear force is extracted for each simulation. These force values are necessary for designing the spring system that controls the ball. The valve offers outflow drainage between 42 and 300 mL/h from min to max pressure points. In terms of force on the ball, it is between 1.5 and 14.7 E-4 N. This includes the pressure points in the supine and the standing position.

### Spring results

**Fig. 8:**
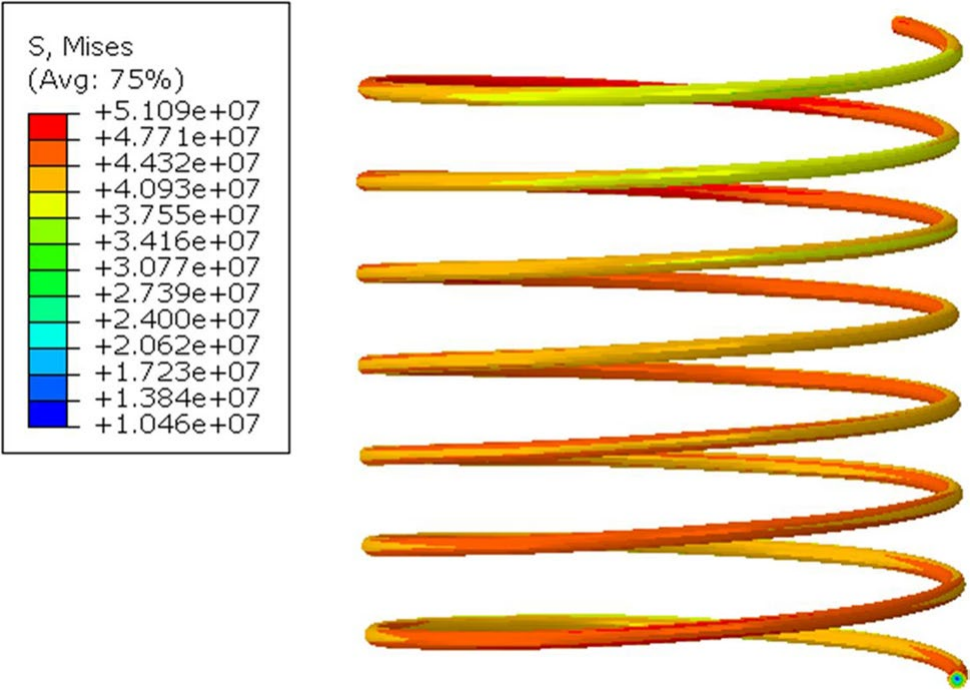
Spring stress results at max operating conditions of 2.22 mm displacement

Figure 8 illustrates the von Mises stress concentration on the spring at 2.22 mm displacement where the maximum stress is 51 MPa. Compared to AZ91E-T6 tensile stress of 275 MPa, it is clear the loading is well below the material limit. The maximum stress is concentrated around the fixed boundary condition (fixed point). The reaction forces (RF) are obtained at the fixed boundary condition. The RFs for the 2.22 and 0.22 mm displacement are 14.6 E-4 N and 1.35 E-4 N, respectively. This is approximately also equal to the force values found in the CFD simulation at max and min operation conditions. This is considered a confirmation of the spring dimensions.

### Ultrasonic element results

#### Modal analysis results

**Fig. 9:**
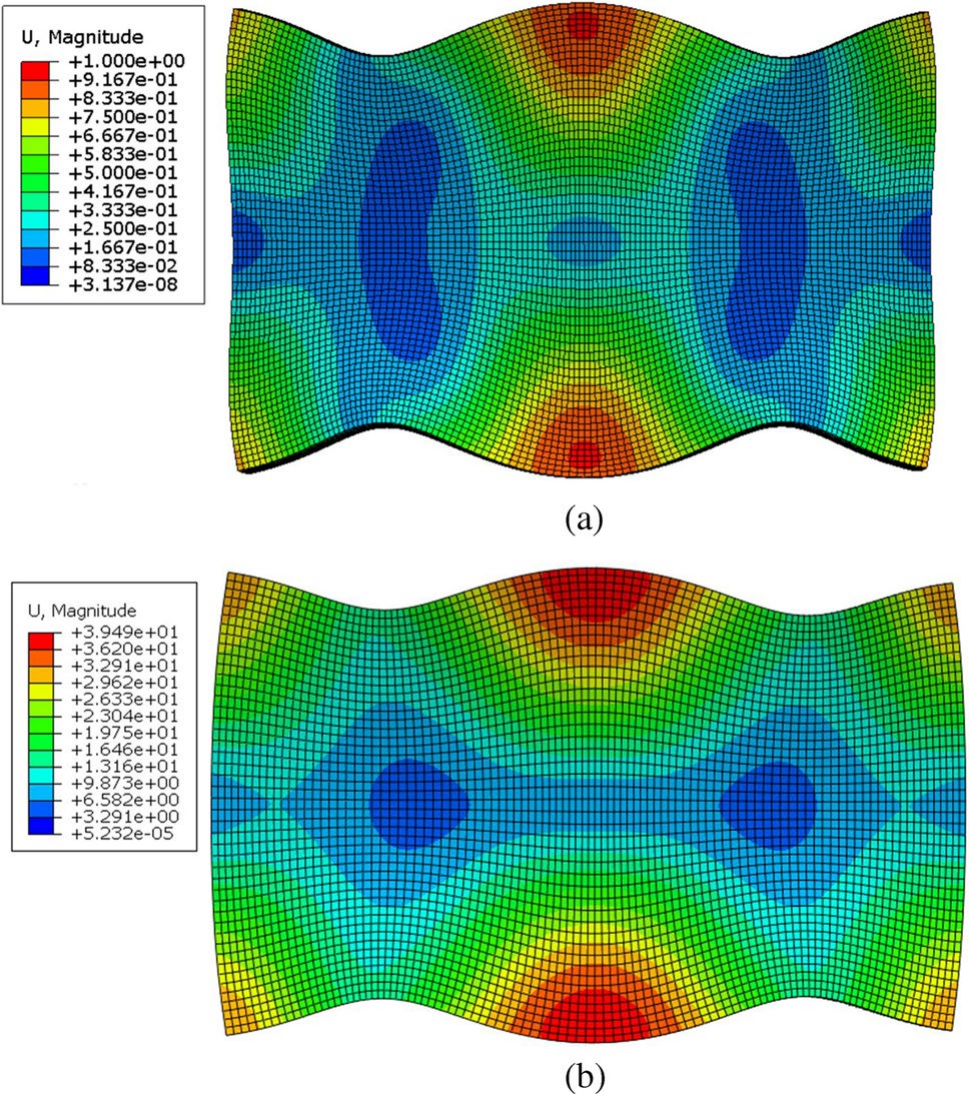
Piezoceramic sheet E(3,1) mode. **a** Resonance frequency of the 13 × 6 × 2 mm sheet at 294.8 kHz. **b** Resonance frequency of the 20 × 7 × 2 mm sheet at 178 kHz

The first E(3,1) modes of the 13 × 6 × 2 mm and the 20 × 7 × 2 mm sheet occur at 294.8 kHz and 178 kHz, respectively. Figure 9 shows the deformation associated with this resonance frequency. The figure showcases the symmetry in deformation in both X and Y axis. This can only be achieved by 2 pairs of standing waves. As this is a frequency analysis, the displacement values shown in the figure and not real. However, relative values are used to compare the simulation results with other studies. No results were obtained for the third 7 × 3 × 1 mm sheet as it proved too small to generate an E(3,1) mode.

**Fig. 10:**
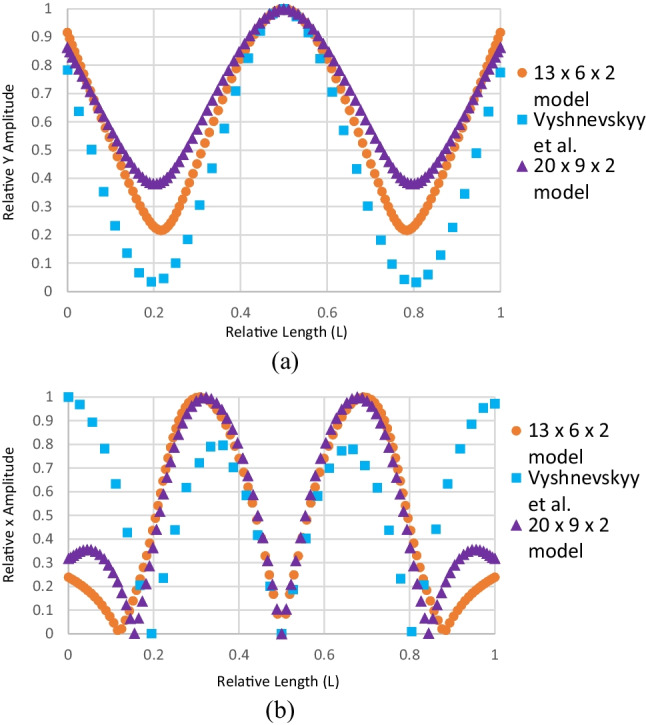
Piezoceramic sheet deformation results. The data taken from Vyshnevskyy et al. [27] are from an experiment and not a simulation. **a** Relative Y amplitude. **b** Relative X amplitude

Figure 10 shows relative displacement on the top plane of the piezoceramic element at $t/2$. The Y relative displacement (amplitude) is compared with that of Vyshnevskyy et al. [27]. For Y amplitude, note that although the two sets of points (Fig. 10a) are not an exact match, their profiles are the same. This is the same case when it comes to the X amplitude (Fig. 10b) where the two sets of points also do not exactly match but have the same profile. This is most likely a result of the difference in size between the two piezoceramic sheets. This further confirms that this is an E(3,1) mode.

#### Dynamic analysis results

**Fig. 11:**
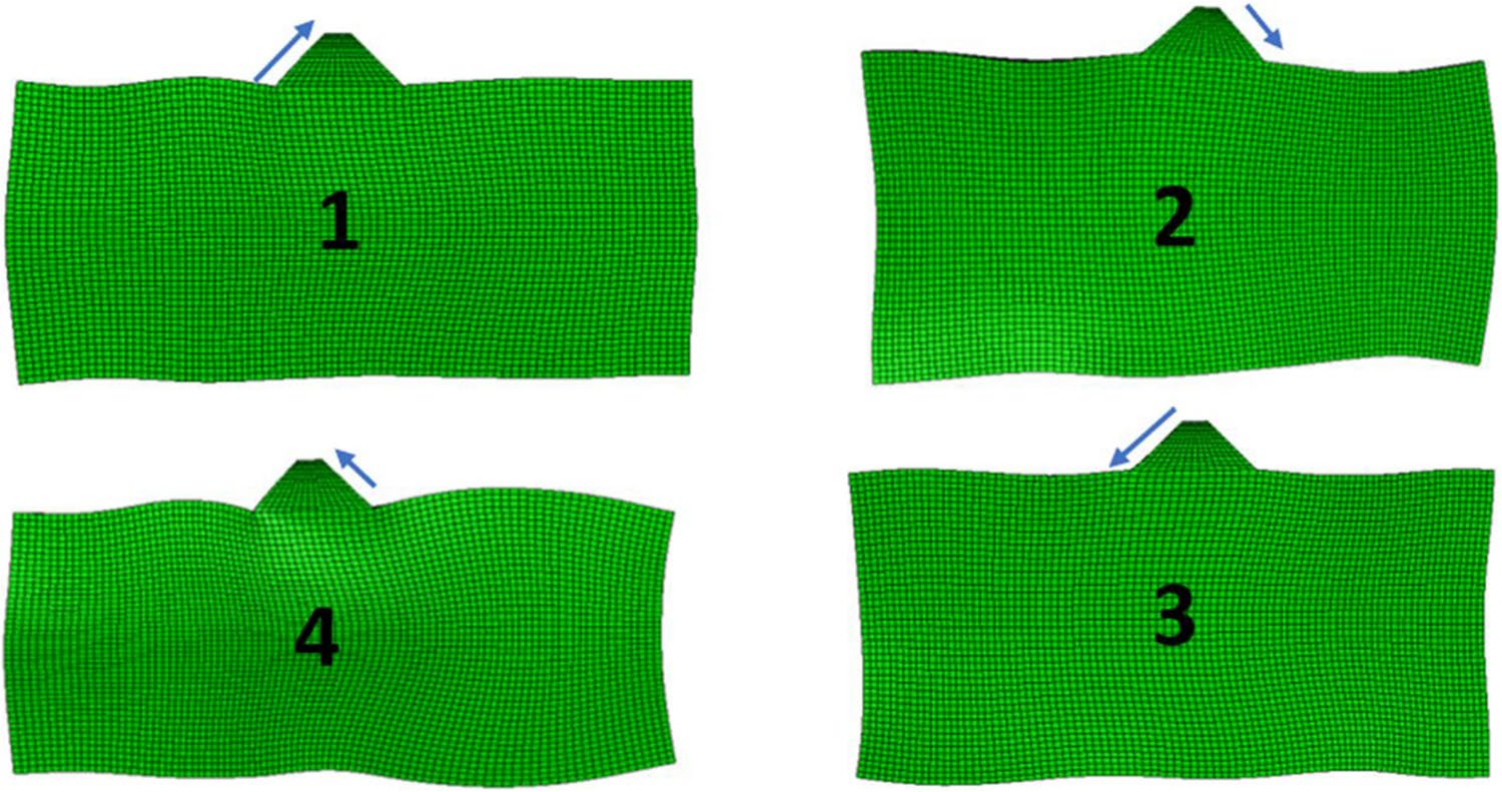
Ultrasonic motor step. Arrows indicate the direction and level of displacement

**Fig. 12:**
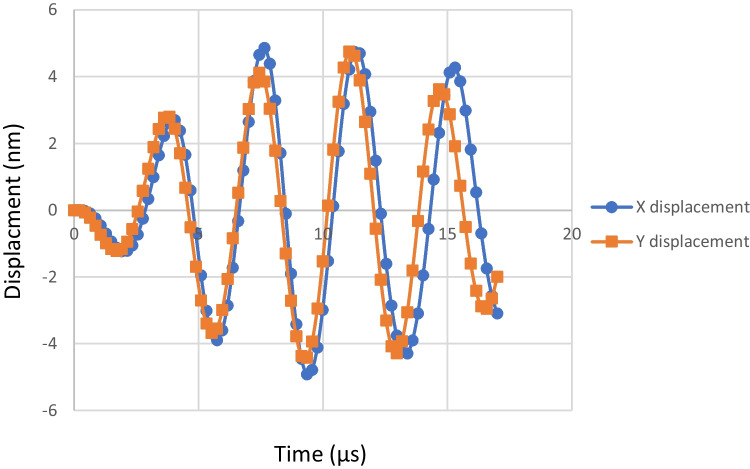
Tip displacement of the 13 × 6 × 2 mm sheet at 294.8 kHz partial excitement

The dynamic simulation ran for 1.7 E-5 s. Figure 11 shows the manner that the tip is affected by the sin wave voltage. The tip is thrust away from the piezo element. It also gains X-axis displacement. The tip goes through one cycle of movement per voltage cycle. Its changes in position are classified into 4 parts. Stator movement is achieved through this 4 parts cycle. First, the tip gains both Y-axis displacement and X-axis displacement. The Y-displacement causes the tip to clamp on the stator while the X-displacement causes the tip to drag the stator along with it as a result of friction. Next, negative Y-displacement and X-displacement are gained. This causes the tip to unclamp and return to its initial position. This is considered a step. Displacement data retrieved from the reference point is shown in Fig. 10. The accurate method to measure the step size is through an experimental setup. However, data retrieved from the reference point indicate the step size. Figure 12 demonstrates X and Y displacement on the tip. The dragging process occurs when the tip is on the positive side of both the X and Y axis. This indicates that the step size is around 4 nm. The motor has a velocity of 1 mm/s. Table 1 presents the number of voltage cycles required to set the opening pressure to each individual pressure point.

**Table 1:** The number of input voltage cycles to the ultrasonic motor required to reach each pressure point. The lowest pressure point with the max length of spring is set as the default starting point (zero point) for these values

ICP		Spring Length (mm)	Piezo element Required cycles		Spring Length (mm)	Piezo element Required cycles
20	Supine position	3.33	4.22E + 04	Standing position	2	5.00E + 05
19		3.39	3.83E + 04		2.05	4.86E + 05
18		3.45	3.42E + 04		2.11	4.72E + 05
17		3.52	3.02E + 04		2.17	4.57E + 05
16		3.58	2.61E + 04		2.23	4.42E + 05
15		3.65	2.19E + 04		2.29	4.27E + 05
14		3.72	1.77E + 04		2.35	4.12E + 05
13		3.79	1.34E + 04		2.41	3.97E + 05
12		3.86	9.02E + 03		2.47	3.82E + 05
11		3.93	4.56E + 03		2.53	3.67E + 05
10		4	initial position		2.6	3.51E + 05

## Discussion

In terms of the CFD analysis, the lack of turbulence makes the simulation results more accurate. Several models were used with different mesh sizes and it was found that the simulation reached mesh independence with the 60,000 mesh size. As for the spring, the magnesium cast is used due to its very low module of rigidity as it can easily deform under low forces which makes it suitable for this application. Furthermore, the cast is biomedically friendly as it does not cause infections when implanted. At max loading, the von Mises stress on the spring is well below the magnesium cast limit. This indicates that the operation range can be increased further than the 20 mmHg upper limit. However, at higher loading conditions, spring stability becomes a point of questioning. For the modal analysis of the ultrasonic element, no results were obtained for the 7 × 3 × 1 mm sheet as there are size constraints when it comes to the generation of standing waves in 2-d bodies. The sheet that provided the required resonance under the lowest frequency is selected. Larger sheets could have been used which will resonate under even lower frequency which will make controlling the ultrasonic element less complicated but for this application small size is necessary. The partial excitations of the selected ultrasonic sheet resulted in a step definition of 4 nm and a speed of 1 mm/s. It must be noted the element will require to be pre-loaded to the stator and that might affect the operation of the element. The extent of the effects can only be observed experimentally. Nevertheless, as the device is operating under low forces, the pre-loading of the element will be minimal and so the effects on its operation.

## Conclusion

There is a consensus in the scientific community that smart shunting is the way forward in order to combat the issues with the current shunting system. A core component of this system is a mechatronic controllable valve that can drain CSF. The valve design proposed in this paper is compact and does not have many moving parts. Design processes were carried out for the three main components of the valve consisting of the fluid compartment, the linear spring, and the ultrasonic element. The use of the ultrasonic element offers a high degree of control since it can offer a 4 nm step definition. It also requires low power due to its low voltage requirement and its offline locking ability. It can be powered using the current lithium batteries used for pacemakers. The combination of the ultrasonic element with the spring means opening pressure values can be set at shorter intervals allowing finer intracranial pressure control. The valve has an operational range of 10–20 mmHg and can operate at a speed of 1 mm/s. The main advantage of the design is that it offers an intrinsic safe mode due to its hybrid nature. The combination of the ultrasonic actuation and the spring system means that if the electronic side of the valve fails the valve can operate as a simple DPV valve. Future work must include experimental validation of this design. Furthermore, the simulated ultrasonic element is under no pre-loading conditions which can affect the operation of the element. However, the preloading will be minimal as the forces exerted from the CSF flow are very low in value.
